# Cefoperazone Sodium Impregnated Polycaprolactone Composite Implant for Osteomyelitis

**DOI:** 10.4103/0250-474X.57285

**Published:** 2009

**Authors:** A. Anand, R. Pundir, C. S. Pandian, S. Saraf, H. Gupta

**Affiliations:** Babu Banarasi Das National Institute of Technology & Management, Lucknow-226 007, India; 1 Department of Pharmaceutics, Faculty of Pharmacy, Jamia Hamdard (Hamdard University), New Delhi-110 062, India

**Keywords:** Calcium sulphate, polycaprolactone, cefoperazone sodium, osteomyelitis, biodegradable implants

## Abstract

The use of local antibiotics from a biodegradable implant for chronic osteomyelitis is an attractive alternative. The implant delivers high antibiotic concentration at tissue levels, obliterates dead space, aids bone repair and does not need to be removed. The purpose of this paper is to develop and evaluate a calcium sulphate and polycaprolactone based composite biodegradable implantable delivery system of cefoperazone sodium. Implants were prepared by modified fabrication technique to avoid solvent use. Interaction studies were carried out to check any incompatibility between ingredients. Prepared implants were evaluated for various *in vitro* parameters like dimensions, hardness, tensile strength, drug release profile and sterility. Morphological changes in pellet before and after drug release were evaluated by scanning electron microscopy. The pellet were also tested for microbiological efficacy and compared with plain drug solution in different concentrations. Developed pellets are regular in shape and size with good tensile strength. The release profile displayed drug levels above MIC continuously up to 2 months. Wide zone of inhibition by pellet against Staph. aureus as compared to drug solution proves its efficacy in treatment of osteomyelitis.

Despite the advances in prophylaxis against infection, chronic osteomyelitis after joint replacement surgery and internal fixation of fractures remains a considerable problem[1–3]. Osteomyelitis is an infection of the bones characterized by pain and nausea, pus formation, edema and warmth over the affected bone and rigid overlying muscles. It is often caused by bacteria usually *Staphylococcus aureu*s. Such infection is often resistant to the parenteral administration of antibiotics. Removal of the implant is often necessary for its control, and usually leads to severe functional disability. The treatment of chronic osteomyelitis includes debridement of the dead infected tissue, obliteration of dead space, osseous repair, adequate soft tissue coverage, and systemic antibiotics. The delivery of antibiotics to bone varies considerably. Oral antibiotics are unpredictable with relatively low bone levels and are infrequently used. Intravenous antibiotics are used commonly in the treatment of chronic osteomyelitis[4]. Six weeks of intravenous antibiotic therapy is necessary for adequate treatment. Although prolonged usage of intravenous antibiotics have significant relapse rate. To supplement systemic antibiotics, local antibiotic delivery has been tried for many years. The primary benefit achieved with local antibiotic delivery vehicle is the ability to obtain extremely high levels of antibiotics at the site of infection[5,6]. This avoids some of the toxicity associated with systemic antibiotics.

A high concentration of antibiotics at the site of the infection would seem to be essential to achieve a cure and on this basis the impregnation of acrylic bone cement with antibiotics was developed[7–10], but main disadvantage in these implants is that they should be removed at the end of the treatment period. Implantable biodegradable polymer systems have a unique advantage that the dosage form need not be removed from the body and thus saves cost and risk for the patient.

Implants are one of the various options for local drug delivery systems. Polymeric materials having slow degradation rates are preferred as implant material. Composite materials are preferred over a single polymer, as implant material had the possibility of variation in physical properties. Various materials other than metal like organic biomaterials, inorganic biomaterials and their composites have been used as artificial bone materials to fill bone defects or to replace bony structures[11]. Composite materials often show an excellent balance between strength and toughness and usually show improved characteristics compared to their separate components[12].

Calcium sulphate was the first material to be used as bone replacement[13]. Here we have used calcium sulphate hemihydrate which is commonly known as plaster of Paris. The plaster has a compressive strength of ~24 MPa[14]. Calcium sulphate is also characterized by its ease of sterilization, complete resorption, biocompatibility and stimulation of bone when in contact with periosteum. Calcium sulphate may act as binder, facilitating healing and preventing loss of grafting material[15]. Moreover it is tissue compatible, so it does not interfere with the healing process. Calcium sulphate has also been used with other materials, such as autogenous bone[16], DFDBA[17] and polymers[18]. Calcium powder acts as a direct source of calcium supply. Generally it completely resorbs within 30-60 day of implantation. Calcium sulphate has approved by FDA for use in fixation of distal radial fracture. Calcium sulphate had been reported as repairing fillers for the treatment of bone defection[19]. Polycaprolactones (PCL) are polyesters related to poly (lactic acid) and poly (glycolic acid) with similar biocompatibility[20]. Polycaprolactones has been used in different biomedical application, such as in scaffolds for tissue engineering of bone and cartilage[21].

Cefoperazone sodium is a second-generation cephalosporin antibiotic which is active against a wide range of Gram-positive and Gram-negative bacteria, including *Staph. aureus*, *Strep. pyogenes, Strep. pneumonia, Neisseria* spp. Cefoperazone sodium interferes with the cell wall synthesis in the bacteria. Therapeutic levels are achieved in CSF only in inflamed conditions. Cefoperazone sodium is used in treating infections of upper and lower respiratory tract, skin and soft tissue, UTI, bone and joint infections and gonococcal infections.

Although an implantable system for ciprofloxacin[22–24] and gatifloxacin[25] has been extensively studied for treatment of bone infection but we have not found any reported literature on composite system of polycarprolactone and calcium sulphate with cefoperazone sodium for treatment of osteomyelitis.

Our present work describes the formulation and evaluation of biodegradable cefoperazone sodium (CFS) impregnated implant for treatment of osteomyelitis. Composite system of calcium sulphate and polycaprolactone was used in its preparation to impart biodegradability and strength.

## MATERIALS AND METHODS

Cefoperazone sodium was received as a gift sample from Torque Pharmaceuticals Pvt. Ltd. Chandigarh, India. Polycaprolactone and calcium sulphate hemihydrate (CSH) was purchased from Sigma Aldrich, USA. All other chemicals and solvents used were purchased from local suppliers and of analytical grade unless mentioned.

### Drug-Polymer Interaction Studies:

The solutions of calcium sulphate hemihydrate, polycaprolactone, and cefoperazone sodium were prepared individually and in combinations and were sterilized using ethylene oxide. The ultraviolet spectra were taken before and after sterilization at 0 h and 24 h using double beam ultraviolet-visible spectrophotometer. Both spectra were compared for any possible change in solution content due to interactions between different ingredients or by sterilization.

### Preparation of implants:

**Fig. 1:**
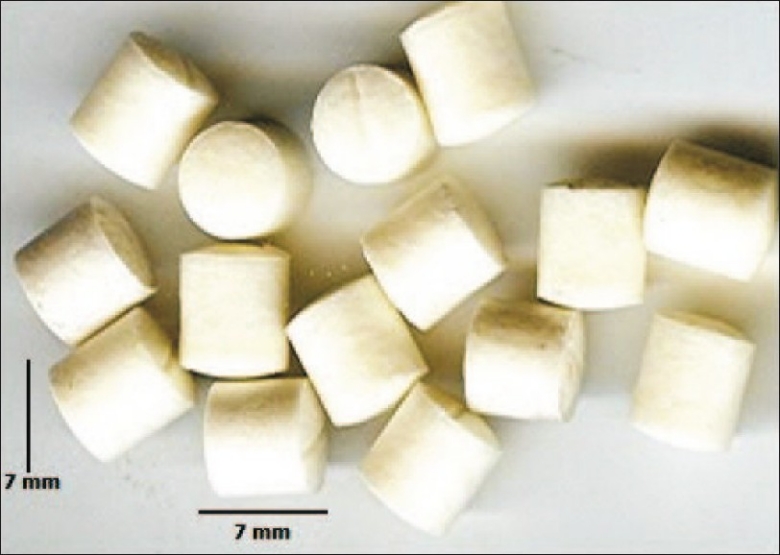
Cefoperazone sodium impregnated calcium sulphatepolycaprolactone composite pellets

Pellets were prepared by a modified fabrication technique. Polycaprolactone polymer, calcium sulphate hemihydrate and cefoperazone sodium was taken in ratio of 1:1:0.75 w/w. Polycaprolactone was first melted and a blend of drug and calcium sulphate hemihydrate were mixed in the molten polymer. The mixture was stirred by a mechanical stirrer for 1 h to ensure proper mixing of the drug with polymer matrix. The composite mixture thus formed was kept for drying. The compact mass was then passed through a sieve No. 16 to form granules. These granules were compressed as pellets in a tablet punching machine, (d-tooling 6 mm, Mini Press, Karnavati, India), fig. 1. The compressed pellets were collected, wrapped in aluminum foils and stored in refrigerator in dry condition till further use. All pellets were sterilized by ethylene oxide sterilization.

### Physicochemical characterization:

Prepared pellets were subjected to physicochemical characterization. Twenty pellets were randomly selected and subjected for dimensional analysis. The diameter and thickness of the pellets were determined by Vernier caliper. For hardness analysis six pellets were randomly selected. Hardness of the pellets was calculated by Monsanto Hardness tester. Rectangular strips of 20×15 mm size were cut and subjected to tensile strength analysis. Tensile strength at breakpoint was measured. Six individual measurements were taken and the data were averaged to obtain a mean value.

### Test for sterility:

Sterility tests were performed for the sterilized pellet to ensure proper sterilization. Three sterilized pelletes were taken and placed in separate petri dish containing 10 ml of culture medium and incubated at 37° for 3 weeks. Petri dishes were observed every week till 3 weeks and evaluated for any growth.

### *In vitro* release profile:

Pellets were taken in the dialysis tube (Sigma Chemicals, USA), which was suspended in beaker at 37±0.5° containing 100 ml artificial simulated body fluid (SBF), pH 7.3 under continuous stirring. The composition of artificial simulated body fluid used was Tris base 6.051g, NaHCO_3_ 0.35g, K_2_HPO_4_ 0.171g, Na_2_SO_4_.10H_2_O 0.16g, KCl 0.224g, CaCl_2_.2H_2_O 0.368g, MgCl_2_. 6H_2_O 0.305g, NaCl 7.996g, 1 N HCl 40 ml and water q.s. 100 ml. Aliquots of medium were withdrawn at different time intervals (24 h duration for first 10 days followed by 72 h duration for next 45 days) and equal volumes of fresh media were added to replace the withdrawn samples. Withdrawn samples were diluted appropriately, and estimated for the drug content by ultraviolet spectrophotometry at 228 nm. Cumulative percent drug released was calculated.

### Scanning electron microscopy studies

Cefoperazone sodium loaded pellets, before and after *in vitro* release, were coated with gold under vacuum and scanning electron micrographs were obtained using a Jeol (JSM- 6400, Japan) scanning electron microscope. Morphological and topographical changes due to antibiotic release were assessed from the micrographs.

### Microbiological Assay:

The cup plate assay was performed on developed pellets as well as on different concentration of plain cefoperazone sodium solution in different Petri plates. Nutrient agar was selected as a nutrient media while *Staphylococcus aureus* MTCC 1430 procured from Institute of Microbial Technology, Chandigarh, India was used as an indicator organism. Previously prepared dilute sample of inoculum was plated over melted agar medium. The agar medium was maintained in a liquid state at 45°. The medium was mixed with inoculum and poured into sterile Petri plates. A cup was made in the plates with the help of cork borer. Three cups numbered 1, 2, 3 were prepared in one plate carrying the antibiotic solution of 0 μg/ml (blank), 15 μg/ml and 30 μg/ml of cefoperazone sodium respectively. These cups were then filled with the drug solution and the plates incubated at 37° for 48 h. Same procedure was followed for pellets by replacing drug solution with pellets. Zone of inhibition was calculated and tabled.

## RESULTS AND DISCUSSION

The interaction studies were carried out to check any possible incompatibility among the formulation ingredients. Ultraviolet spectra of the ingredients before and after sterilization at 0 h and 24 h were identical. No additional peak was observed which confirms that the formulation ingredients were compatible and no physicochemical reactions took place due to sterilization by ethylene oxide.

Pellets formed were shown in fig. 1. All pellets formed were of uniform shape and size (diameter 6.81±0.027, thickness 7.01±0.014). The measured tensile strength and hardness of pellets was found to be 1 N and 9.33±0.68 respectively indicating reasonable mechanical properties of the prepared implant. Sterilization of the packaged product was done by gaseous sterilization using ethylene oxide and test for sterility was performed on sterilized packaging according to IP 1996 standards. No microbial growth/microbial contamination were observed up to 14 days of incubation. Hence the formulation passed the sterility test.

**Fig. 2:**
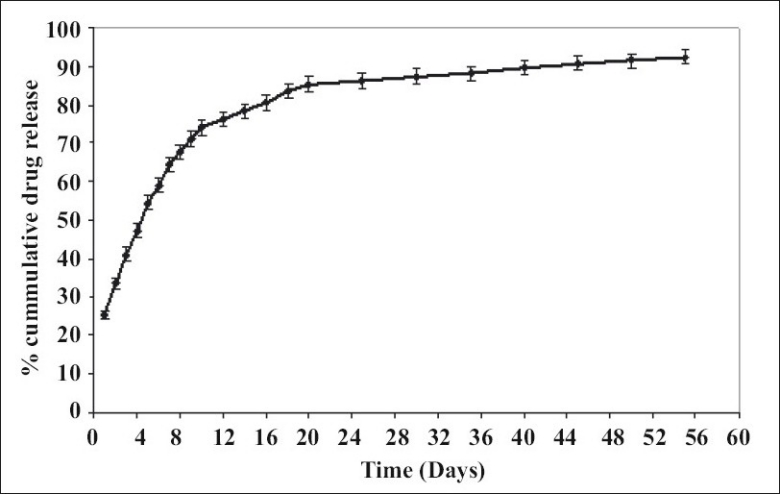
*In vitro* release behavior of cefoperazone sodium implants

*In vitro* release behavior of formulations was determined in artificial simulated body fluid. The release profile of developed cefoperazone composite implant revealed that 54.4% of drug was released in first 5 days. Then there is a continuous release of 74.1% drug in 10 days followed by slow release till 55 days. The release profile was characterized by an initial burst followed by a second stage of gradual delivery of 92.4% over 55 days (fig. 2). Burst release is a phenomenon frequently associated with polymeric controlled drug delivery systems. Maintaining a high antibiotic concentration at the site of infection is highly appropriate for treating multibacterial infection and consequently increases the effectiveness of treatment.

**Fig. 3:**
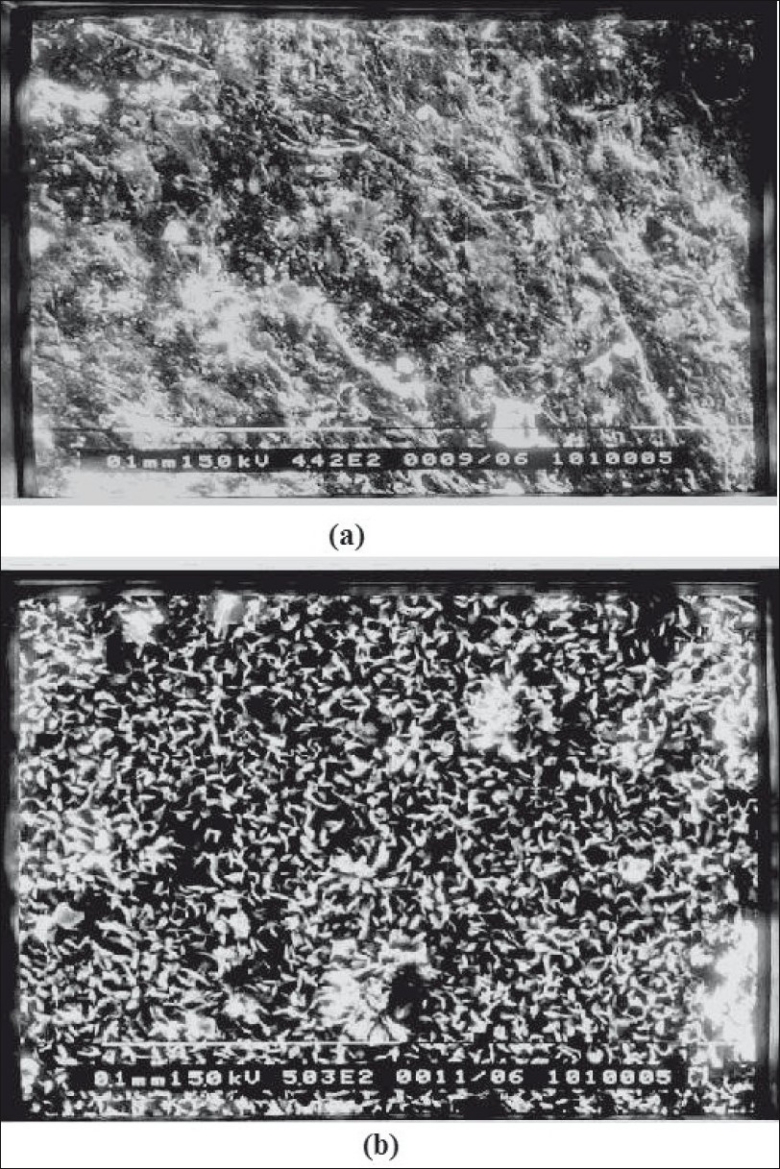
Scanning electron micrographs of cefoperazone sodium pellets. Scanning electron micrographs showing the surface morphology of cefoperazone sodium pellets (a) before and (b) after *in vitro* release study.

In general, rods displayed very similar texture and morphology regardless of the polymer and antibiotic type used. Cefoperazone sodium loaded pellets were examined before and after 15 days in the release medium. It was observed that before release, the antibiotic crystals on the surface of the rods were partially exposed (fig. 3a) and surface was smooth as compared to SEM micrograph of pellet after drug release. When the drug-loaded pellets were placed in the aqueous environment, pores were created on the exposed part due to removal of drug particles through dissolution. These pores led to water penetration into the pellet and caused further drug dissolution. (fig. 3b). Voids generated reveals uniform drug distribution and release due to diffusion from pellets.

**Fig. 4:**
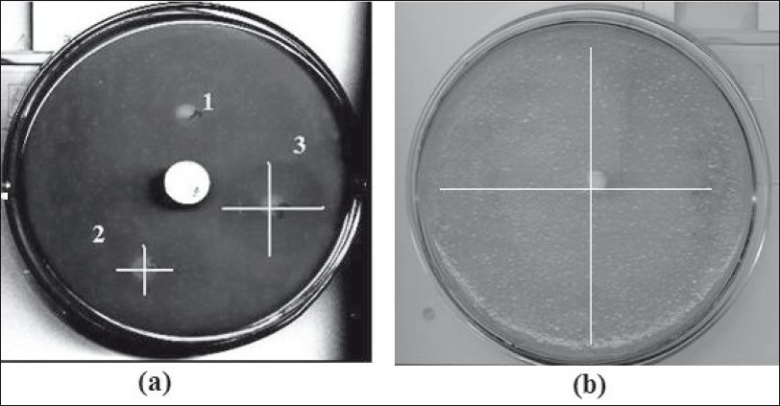
Zone of inhibition in cup plate assay. (a) Different drug concentration (1) Blank Solution (2) 15 μg/ml concentration (3) 30 μg/ml concentration (b) Pellet formulation

Cup plate assay of the plain cefoperazone sodium solution showed inhibitory activity of this drug against the *S. aureus*. Zone of inhibition is concentration dependent as clearly seen, Table 1. The cup carrying 30 μg/ml (fig. 4a, 3) concentration shows a bigger zone of inhibition as compared to 15 μg/ml concentration (fig. 4a, 2). Diffusion of drug from the pellets also showed a clear zone of inhibition. A large zone of inhibition (almost covering entire plate) was observed at 24 h in plate carrying the intact pellet, fig. 4b. It indicates efficacy of developed pellets in comparison to simple drug solution.

**TABLE 1:** ZONE OF INHIBITION OF DIFFERENT CONCENTRATION OF CEFOPERAZONE SODIUM SOLUTION AND PELLETS

Antibiotic medium	Concentration (μg/ml)	Zone of Inhibition (mm)
Cefoperazone sodium solution	15	12.33±1.43
Cefoperazone sodium solution	30	25.67±1.53
Cefoperazone sodium pellet	-	35.37±1.1

Values are expressed as mean±SD of *n* = 6 observations.

Till date various materials have been tested for the treatment of osteomyelitis either as a single or as a composite material that can release drug over a period of one month. The developed calcium sulphate and polycaprolactone biodegradable composite prolonged the drug release up to two months while maintaining therapeutic levels above the MIC of cefoperazone sodium against *S. aureus*. These pellets are found suitable for treatment of osteomyelitis and can go up to clinical level.
